# Case Report: Stereotactic body radiation treatment for immunotherapy escaped oligometastatic progression in cutaneous melanoma and merkel cell carcinoma

**DOI:** 10.3389/fonc.2023.1276729

**Published:** 2023-09-20

**Authors:** Karam Khaddour, Alice Zhou, Omar Butt, Jiayi Huang, George Ansstas

**Affiliations:** ^1^ Division of Hematology and Oncology, University of Illinois Chicago, Chicago, IL, United States; ^2^ Department of Medicine, Division of Medical Oncology, Washington University in Saint Louis- Division of Medical Oncology, Saint Louis, MO, United States; ^3^ Department of Radiation Oncology, Washington University School of Medicine, Saint Louis, MO, United States

**Keywords:** melanoma, merkel cell carcinoma, oligometastatic progression, stereotactic body radiation therapy, immunotherapy, CtDNA

## Abstract

Oligometastatic progression represents a unique manifestation of tumor immune-escape that can lead to disease progression during treatment with immune checkpoint inhibitor (ICI). The diagnosis and further optimal management of oligometastatic progression through ICI remains unclear. Diagnostic challenges include practical limitations due to the anatomical sites of oligometastatic progression, such as the para-aortic region, where traditional tissue biopsy carries high risk, and circulating-tumor DNA (ctDNA) could aid in diagnosis and disease monitoring as a supplement to surveillance imaging. In this report, we describe two cases of one patient with metastatic melanoma and the other with metastatic Merkel cell carcinoma (MCC) who were treated with ICI and later developed localized resistance due to oligometastatic progression. We further highlight our experience using stereotactic body radiation therapy (SBRT) as a salvage approach to treat the oligometastatic progression. In addition, we describe the temporal and dynamic relationship of circulating-tumor DNA (ctDNA) prior to, during and after SBRT, which highly suggested the diagnosis without obtaining a histological specimen. Our cases highlight a potential role for SBRT in the management of oligometastatic progression. However, large prospective trials are essential to confirm the utility of this approach.

## Background

Acquired resistance to immune checkpoint inhibitors (ICI) can develop during the treatment of skin cancers including metastatic melanoma and Merkel cell carcinoma (MCC), leading to treatment failure (1, 2). Oligometastatic progression represents a unique manifestation of immune escape during treatment with ICI with the potential for leveraging salvage therapies. There is a lack of knowledge on best treatment approach in these circumstances given lack of available strong evidence (1, 2). Patient-centered discussion in a multidisciplinary setting and participation in clinical trials when available are considered best practice. Of note, one approach of management includes continuation of systemic therapy if there is durable response outside of the oligometastatic progressive site, combined with a localized salvage treatment to the immune-escaped metastatic lesion. Surgical or local radiation therapy are considered on individual basis, although there remains lack of consensus opinion and data to support this concept (3). This is further complicated by the difficulty in the interpretation of clear progression on imaging in the era of ICI where pseudoprogression is common, representing a challenge to appropriate medical decision making. To this end, circulating-tumor DNA (ctDNA) may serve as a supplemental non-invasive tool to provide information on oligometastatic tumor progression and the dynamic changes during local treatment.

In this report, we describe two cases of metastatic melanoma and MCC that were associated with oligometastatic progression during treatment with ICI despite achieving a complete response systemically elsewhere. These oligometastatic lesions were not accessible for tissue biopsy. Our management approach included salvage local stereotactic body radiation therapy (SBRT) with continuation of systemic ICI. We report the outcomes of these two cases, and the dynamic interplay between ctDNA alterations prior to, and after SBRT, which confirmed metastatic involvement without the need for a histological specimen. These results are intriguing and provide the basis for further investigation in larger cohorts to assess the efficacy and safety of salvage SBRT in the management of immune-escaped oligometastatic progression.

## Case presentation

### Case 1

**Figure 1:**
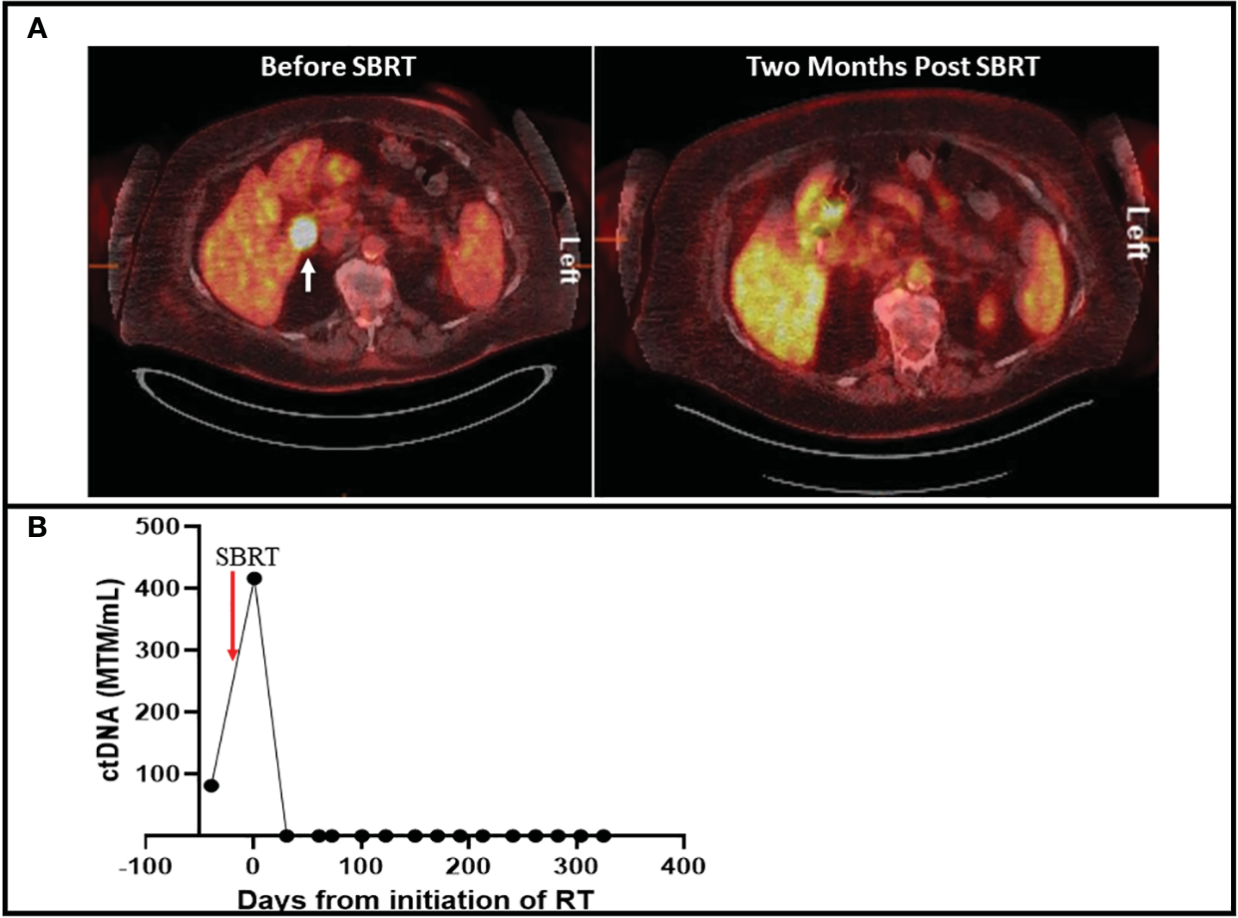
**(A)** 18 Fludeoxyglucose PET-CT with cross sectional imaging on the left demonstrating an FDG avid portocaval lesion with a standardized uptake value of 18.7 (white arrow). Right figure demonstrates resolution of the FDG-avid lesion after SBRT. **(B)** Timeline of ctDNA changes during and after SBRT. Levels of ctDNA are quantified by mean tumor mutation per milliliter (MTM/mL).

A 79-year-old female presented with a new nodular erythematous lesion on the right chest wall. Excisional biopsy demonstrated MCC. Positron Emission Tomography- Computed Tomography (PET-CT) was negative for metastatic disease. The patient underwent wide local excision and sentinel lymph node biopsy (SLNB) with pathology demonstrating negative margins and negative SLNB. After 7 months of surveillance, PET-CT demonstrated new liver lesions, which were biopsy proven to be MCC. The patient started pembrolizumab and had complete response within 6 months of initiating treatment. Subsequent PET-CT after 9 months demonstrated an interval enlargement of a portocaval lymph node measuring 3.0 x 2.2 cm with a standardized uptake value (SUV) of 18.7 (**Figure 1A**). A personalized patient-specific tumor-informed assay (Signatera™) was used to quantify the tumor mutation molecule per milliliter to detect circulating tumor DNA (ctDNA). Measurement of ctDNA demonstrated 42.33 mean tumor molecules per milliliter (MTM/mL) (**Supplementary File-1**). The patient underwent stereotactic body radiation therapy (SBRT) of 35 Gray in 5 fractions directed to the portocaval nodal basin. Monitoring of ctDNA during radiation treatment demonstrated increased levels followed by undetectable levels (**Figure 1B**). Repeat PET-CT after 2 months of SBRT demonstrated complete resolution of excessive FDG avid uptake in the portocaval lymph node (**Figure 1A**). The patient continued pembrolizumab without observed side effects. Surveillance imaging alternating with ctDNA monitoring after 10 months of SBRT continued to show no evidence of disease.

### Case 2

**Figure 2:**
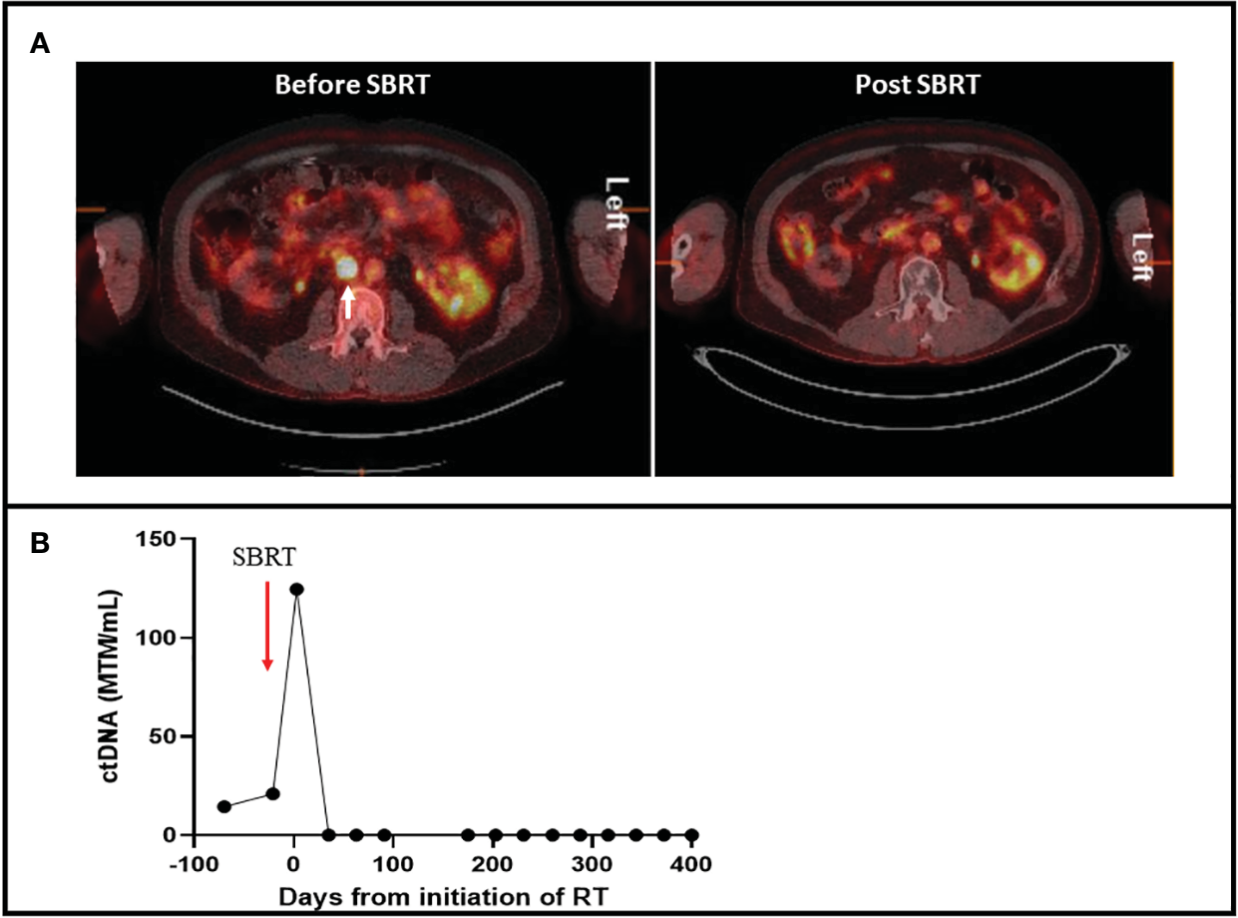
**(A)** 18 Fludeoxyglucose PET-CT with cross sectional imaging on the left demonstrating an FDG avid aortocaval lesion with a standardized uptake value of 21.8 (white arrow). Right figure demonstrates resolution of the FDG-avid lesion after SBRT. **(B)** Timeline of ctDNA changes during and after SBRT. Levels of ctDNA are quantified by mean tumor mutation per milliliter (MTM/mL).

A 65-year-old male was diagnosed with *BRAF^V600E^* metastatic intracranial melanoma. A craniotomy was performed with gross total resection followed by hypofractionated stereotactic radiosurgery (SRS) of the resected tumor bed. The patient then commenced ipilimumab and nivolumab for four cycles followed by maintenance nivolumab. Surveillance imaging demonstrated recurrence of the intracranial mass after 4 months of ICI therapy. He underwent repeat craniotomy and whole brain radiation. BRAF-MEK inhibitors were attempted but the patient had intolerable side effects necessitating discontinuation. He resumed maintenance nivolumab, which was continued for 3 years at which time PET-CT demonstrated a FDG avid aortocaval lymph node measuring 1.7 x 1.6 cm with SUV of 21.8 (**Figure 2A**). Tissue biopsy was not possible given the location of the lymph node. However, tumor-informed ctDNA was positive and quantified at 20.92 MTM/mL (**Figure 2B**). After a consensus quorum at multi-disciplinary tumor boards, the patient was treated with SBRT of 50 Gray in 5 fractions. Measurement of ctDNA after one day of initiating SBRT increased to 124.55 MTM/mL. The patient continued nivolumab without observed side effects. Further longitudinal monitoring of ctDNA was undetectable for 10 months (**Figure 2B**), and there was no evidence of disease recurrence on imaging surveillance.

## Discussion

Immune checkpoint inhibitors (ICI) represent the standard of care for the management of metastatic melanoma and advanced non-melanoma cutaneous malignancies. This is highlighted by the durable response ranging from 5.9 to 34.5+ months, and the high overall response rate (56%) associated with pembrolizumab treatment in recurrent locally advanced and metastatic MCC (4, 5). Similarly, the treatment backbone of metastatic melanoma consists of ICI as a first line treatment due to the high response rates and prolonged overall survival (1). However, a substantial proportion of skin cancer patients treated with ICI develop progressive disease during the course of their treatment. Oligometastatic progression represents a unique biological and clinical entity in which cancer progression is limited to ≤ 5 lesions, and confined to a small region or one organ (3). The occurrence of oligometastatic progression during treatment of metastatic skin cancer is common. For example, in the CheckMate-067, approximately one-third of patients with metastatic melanoma treated with ipilimumab and nivolumab developed progressive disease of which 42% had metastatic progression at least in one site with the lymph nodes being the most involved site at progression (6).

There is a lack of data to support best treatment approach for patients with unique patterns of progression such as oligometastatic disease progression (3). Several studies highlighted a potential role for curative local control with surgery or radiation therapy of the oligometastatic site in different cancers (3). For example, in colorectal cancer and non-small cell lung cancer, surgical resection or the use of SBRT was considered an acceptable option in both synchronous and metachronous progressive oligometastatic disease (3, 7–9). However, most of the evidence is weak-to moderate given that it was derived from non-randomized prospective or retrospective studies (3). In regards to immunotherapy, some patients could develop metachronous oligometastatic disease despite persistent durable complete or partial response elsewhere. These unique cases are challenging to physicians and patients and raise several questions regarding the efficacy of ICI continuation with a salvage local treatment approach aimed at the oligometastatic progressive site, or the option of ICI discontinuation and switching systemic therapy. This decision depends on institution experience, multidisciplinary panel recommendations, and patient preference. National guidelines provide salvage local treatment as an option for consideration (1). Most of this evidence is derived from retrospective studies conducted in the pre-immunotherapy era, which did not focus on oligometastatic progression (10). One recent study provided some insight on the use of local RT with immunotherapy in melanoma. This study included a cohort of 1675 patients with extracranial metastatic melanoma who were receiving ICI and were treated with local RT. The investigators found no overall survival benefit in patients who received RT with immunotherapy versus those who received immunotherapy alone in a multivariate analysis (11). However, this retrospective study did not focus on patients who develop oligo-progression during treatment. Similarly in MCC, the addition of SBRT to ipilimumab and nivolumab did not improve overall survival in a randomized controlled trial (12). Therefore, the role of SBRT in immune-escaped oligometastatic progression remains unexplored. Another challenge in the evaluation of oligometastatic progression arises when the suspicious lesion on imaging is located in an anatomical site where it is difficult to obtain a tissue specimen for histopathological examination. In these situations, clinical manifestations as well as imaging remain the only available tools to suggest a possibility of disease progression, and to warrant further treatment. Despite the reliability of imaging modalities in the diagnosis of tumor progression in melanoma and MCC, false positive results can occur leading to unnecessary invasive diagnostic testing or premature treatment discontinuation as well as patient anxiety (13).

The current available evidence of RT with immunotherapy is focused on the safety and efficacy of using these modalities in combination or a sequential fashion (14). This stems from data supporting the role of RT in reprogramming the tumor microenvironment leading to improved response to ICI and enhancing an abscopal effect (15, 16). A major unanswered question remains on the efficacy of salvage local RT in patients with metastatic skin cancer who respond initially to immunotherapy and later develop oligometastatic progression. The two patients presented here had complete responses to ICI that were maintained but then developed disease progression in one isolated site. We used SBRT as a salvage approach which was aimed at the oligometastatic sites and continued immunotherapy without observed immune related adverse events or disease recurrence.

Moreover, the two patients who had durable response to ICI, developed potential oligometastatic progression on surveillance imaging that were not biopsied. We sought to use ctDNA as a complementary tool to confirm malignant involvement of the locally treated site through longitudinal monitoring using a tumor-specific patient-informed ctDNA platform. We observed a rise in patients’ ctDNA from baseline during treatment with SBRT to the involved site. This highly suggested that the treated sites were likely malignant. Elevation of ctDNA levels has been described to occur after surgery and RT due to tumor necrosis and release of fragmented tumor DNA into the peripheral blood (17, 18). Longitudinal surveillance using a combined modality of PET-CT alternating with ctDNA demonstrated no evidence of disease progression after SBRT and ICI in our patients. Of importance, the use of ctDNA for longitudinal monitoring in skin cancer has only been reported in retrospective studies and case-series and lacks strong evidence to support its use in clinical practice (19, 20).

In conclusion, our report is the first to our knowledge to describe the clinical outcomes of two patients with metastatic MCC and melanoma who were treated with immunotherapy and developed oligometastatic progression despite durable response to ICI elsewhere. These patients were treated with SBRT targeting the immune-escaped lesion and continued ICI with no evidence of disease recurrence on imaging and ctDNA monitoring. Our report is important for the following aspects: 1) it highlights a potential role of salvage SBRT in cases where durable response to immunotherapy is maintained outside of a progressive oligometastatic site; 2) it provides an illustration of the dynamic interplay between ctDNA and SBRT which can highly suggest malignant involvement of the suspicious site in cases were biopsies are difficult to obtain; and 3) it suggests a role for ctDNA as a supplemental minimally-invasive tool to imaging for surveillance. This report has its limitations given the small number of patients, retrospective nature of the observation, and the absence of a comparative arm. These results are intriguing and provide a basis for further research in a large prospective cohort.

## Data availability statement

Data was acquired through patient chart review using Washington University in Saint Louis medical records.

## Ethics statement

Written informed consent was obtained from the participant/patient(s) for the publication of this case report. A copy of the written consent form is available at the editorial office for this journal.

## Author contributions

KK: Conceptualization, Data curation, Investigation, Writing – original draft, Writing – review & editing. AZ: Writing – original draft, Writing – review & editing. OB: Writing – review & editing. JH: Writing – review & editing. GA: Conceptualization, Supervision, Writing – original draft, Writing – review & editing.
