# Supplementary material for: Case Report: Stereotactic body radiation treatment for immunotherapy escaped oligometastatic progression in cutaneous melanoma and merkel cell carcinoma
Source: Front Oncol. 2023 Sep 20;13:1276729. doi: 10.3389/fonc.2023.1276729 (PMC10547860; doi:10.3389/fonc.2023.1276729)
Supplement: Supplementary file 1 [file DataSheet_1.docx]

**Method of ctDNA Assay**

ctDNA was detected and quantified using multiplex (m)PCR-next generation sequencing (NGS)-based ctDNA assay based ctDNA assay (Signatera™), which has been previously described in detail (1). Plasma samples were collected periodically in an alternating fashion with radiological imaging. Due to the retrospective nature of data collection, there were no fixed interval time points at which patients had ctDNA testing performed. We collected two tubes of whole blood (~20 ml) in Streck Cell-Free DNA BCTs for each patient at each time point. All blood samples had plasma isolated within 9 days of collection by single-spin centrifugation of the blood at 22°C, for 30 min at 3,220g, and was stored at 4°C until further use. Cell-free DNA extraction from plasma samples was performed using QIAsymphony DSP Circulating DNA Kit. Tumor tissue was collected from all patients as fresh frozen or as formalin-fixed and paraffin-embedded tissue (FFPE). Cell-free DNA was extracted using the Puregene DNA purification kit (Gentra Systems) or using the QiAmp DNA FFPE tissue kit (Qiagen). To design the ctDNA assay for each patient, WES was performed on biopsied tumor tissue, along with a matched-normal whole blood sample. Sequencing results were analyzed using Natera’s proprietary tissue variant calling pipeline, and 16 highly ranked tumor-specific somatic, clonal, single-nucleotide variants (SNVs) were selected for mPCR primer design for each personalized ctDNA assay. Plasma samples were later collected and were subjected to cfDNA extraction, followed by cfDNA library preparation. The cfDNA was end-repaired, A-tailed, and ligated with custom adapters, followed by amplification and purified using Ampure XP beads (Agencourt/Beckman Coulter). A proprietary mPCR methodology was used to run patient-specific assays. The mPCR product is then barcoded, pooled, and sequenced on the Illumina HiSeq 2500. A plasma sample was considered to be ctDNA-positive if at least two out of the 16 SNVs were detected. ctDNA levels were quantified in mean tumor molecules per milliliter of plasma (MTM/ml) (2).

**Reference**

1. Reinert T, Henriksen TV, Christensen E, Sharma S, Salari R, Sethi H, Knudsen M, Nordentoft I, Wu HT, Tin AS, Heilskov Rasmussen M, Vang S, Shchegrova S, Frydendahl Boll Johansen A, Srinivasan R, Assaf Z, Balcioglu M, Olson A, Dashner S, Hafez D, Navarro S, Goel S, Rabinowitz M, Billings P, Sigurjonsson S, Dyrskjøt L, Swenerton R, Aleshin A, Laurberg S, Husted Madsen A, Kannerup AS, Stribolt K, Palmelund Krag S, Iversen LH, Gotschalck Sunesen K, Lin CJ, Zimmermann BG, Lindbjerg Andersen C. Analysis of Plasma Cell-Free DNA by Ultradeep Sequencing in Patients With Stages I to III Colorectal Cancer. JAMA Oncol. 2019 Aug 1;5(8):1124-1131. doi: 10.1001/jamaoncol.2019.0528. Erratum in: JAMA Oncol. 2019 Jun 13;: PMID: 31070691; PMCID: PMC6512280.
2. Abbosh C, Birkbak NJ, Wilson GA, Jamal-Hanjani M, Constantin T, Salari R, Le Quesne J, Moore DA, Veeriah S, Rosenthal R, Marafioti T, Kirkizlar E, Watkins TBK, McGranahan N, Ward S, Martinson L, Riley J, Fraioli F, Al Bakir M, Grönroos E, Zambrana F, Endozo R, Bi WL, Fennessy FM, Sponer N, Johnson D, Laycock J, Shafi S, Czyzewska-Khan J, Rowan A, Chambers T, Matthews N, Turajlic S, Hiley C, Lee SM, Forster MD, Ahmad T, Falzon M, Borg E, Lawrence D, Hayward M, Kolvekar S, Panagiotopoulos N, Janes SM, Thakrar R, Ahmed A, Blackhall F, Summers Y, Hafez D, Naik A, Ganguly A, Kareht S, Shah R, Joseph L, Marie Quinn A, Crosbie PA, Naidu B, Middleton G, Langman G, Trotter S, Nicolson M, Remmen H, Kerr K, Chetty M, Gomersall L, Fennell DA, Nakas A, Rathinam S, Anand G, Khan S, Russell P, Ezhil V, Ismail B, Irvin-Sellers M, Prakash V, Lester JF, Kornaszewska M, Attanoos R, Adams H, Davies H, Oukrif D, Akarca AU, Hartley JA, Lowe HL, Lock S, Iles N, Bell H, Ngai Y, Elgar G, Szallasi Z, Schwarz RF, Herrero J, Stewart A, Quezada SA, Peggs KS, Van Loo P, Dive C, Lin CJ, Rabinowitz M, Aerts HJWL, Hackshaw A, Shaw JA, Zimmermann BG; TRACERx consortium; PEACE consortium; Swanton C. Phylogenetic ctDNA analysis depicts early-stage lung cancer evolution. Nature. 2017 Apr 26;545(7655):446-451. doi: 10.1038/nature22364. Erratum in: Nature. 2017 Dec 20;: PMID: 28445469; PMCID: PMC5812436.
